# Application of Mendelian randomization analysis to explore causal associations of aspirin use with bone mineral density and risk of fracture

**DOI:** 10.1186/s41065-024-00359-3

**Published:** 2025-01-07

**Authors:** Qi-Pei Liu

**Affiliations:** Guangzhou University of Chinese Medicine, Guangzhou, 510405 China

**Keywords:** Mendelian randomization, Aspirin, Osteoporosis, Bone mineral density, Fracture, Causality

## Abstract

**Objective:**

Previous observational studies on the association between aspirin use, bone mineral density (BMD), and fracture risk have yielded controversial results. This study explored the causal relationship between aspirin use, BMD, and fracture risk using Mendelian randomization (MR).

**Methods:**

Summary data for aspirin use and BMD of five different body parts (femoral neck, lumbar spine, forearm, heel, and ultra distal forearm) and fractures were obtained from the integrative epidemiology unit open genome-wide association studies database for bidirectional MR analysis. An appropriate model was chosen based on Cochran's Q test, with inverse variance-weighted as the primary method for MR analysis, supplemented by the weighted-median and MR-Egger methods. MR-Egger and MR-PRESSO were used to test for horizontal pleiotropy and exclude significant outliers that could bias the results. Various sensitivity analyses, including leave-one-out analysis, were conducted to ensure the robustness of the findings.

**Results:**

Aspirin use significantly increased lumbar spine BMD (odds ratio [OR] = 4.660; 95% confidence interval [CI]: 1.365–15.906; *P* = 0.014). No significant causal association was found between aspirin use and fracture risk (beta = 59.951; 95% CI: -265.189–385.091; *P* = 0.718). No significant reverse causality was observed.

**Conclusion:**

This study indicates that aspirin use does not significantly affect fracture risk but has a significant protective effect on lumbar spine BMD, revealing a potential benefit of aspirin against osteoporosis.

**Supplementary Information:**

The online version contains supplementary material available at 10.1186/s41065-024-00359-3.

## Introduction

Osteoporosis is a chronic bone metabolic disease caused by disruption of the balance between bone resorption and bone formation, leading to decreased bone mineral density (BMD) and destruction of the bone microstructure [1]. The prevalence of osteoporosis is gradually increasing with the aging population, with approximately 35% of elderly men and 13% of elderly women affected globally [2]. The annual economic loss due to fracture caused by unprevented and untreated osteoporosis is substantial in the UK and the US, amounting to approximately £4 billion and $1.8 billion respectively [3]. Moreover, increasing evidence suggests a potential association between osteoporosis and cardiovascular diseases [4].

Aspirin (acetylsalicylic acid) is widely used for its antipyretic and anti-inflammatory properties because it irreversibly inhibits cyclooxygenase (COX) enzymes, which are precursors of prostaglandins and thromboxanes [5]. Owing to its high efficacy in inhibiting COX-1, it has subsequently become a common medication in the cardiovascular field owing to its antiplatelet characteristics [6]. A study has shown that aspirin can prevent and treat osteoporosis by promoting osteoblast activity and inhibiting osteoclast activity through dual regulatory mechanisms [7]. However, some studies suggest that aspirin may negatively regulate bone mineral density, which is attributed to the promotion of nitric oxide release and the balance of prostaglandins (PGs) in the body [8, 9].

Previous observational studies on the association between aspirin use and bone mineral density in humans have been controversial and contradictory, questioning whether aspirin has a protective effect [10–14], poses a risk [15], or is unrelated to BMD [16]. Furthermore, observational studies have not reached a consensus regarding the relationship between aspirin use and fracture risk [11, 14, 17, 18]. Observational studies are inevitably affected by reverse causality and potential confounding factors, leading to doubts about the credibility of their conclusions.

The Mendelian randomization (MR) method has become a widely favored epidemiological approach because of its resistance to confounding by external environmental factors [19]. Single-nucleotide polymorphisms (SNPs) strongly associated with exposure are used as instrumental variables (IVs) to explore causal relationships between exposure and outcome [20]. Similar to random assignment in randomized controlled trials, MR minimizes potential confounding factors and reverse causality by utilizing the random allocation of genes from parents to offspring [21, 22]. Therefore, this study employed the MR method to investigate the causal relationship between aspirin use, bone mineral density, and fracture risk.

## Materials and methods

Summary data for aspirin use and BMD of five different body parts (femoral neck, lumbar spine, forearm, heel, and ultra distal forearm) and fractures were obtained from the integrative epidemiology unit open genome-wide association studies database for bidirectional MR analysis. An appropriate model was chosen based on Cochran’s Q test, with inverse variance-weighted as the primary method for MR analysis, supplemented by the weighted-median and MR-Egger methods. MR-Egger and MR-PRESSO were used to test for horizontal pleiotropy and exclude significant outliers that could bias the results. Various sensitivity analyses, including leave-one-out analysis, were conducted to ensure the robustness of the findings.

### Study design

**Fig. 1:**
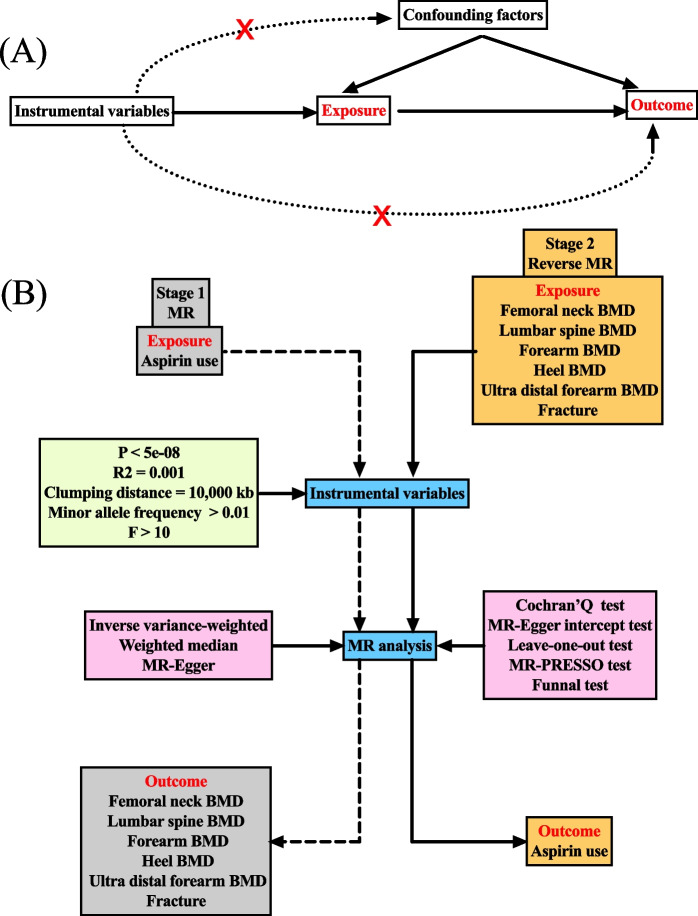
Flowchart of the study design

Bidirectional MR analysis was conducted to explore the causal relationship between aspirin use, bone mineral density, and fracture risk. Three fundamental assumptions were met in the process: first, SNPs significantly associated with exposure were selected as IVs; second, SNPs should be independent of confounding factors and outcomes; and third, SNPs should affect outcomes solely through exposure [23]. The flowchart of the study design is shown in Fig. 1.

### Data source

All data involved in this study were available from the publicly accessible integrative epidemiology unit (IEU) open genome-wide association studies (OpenGWAS) database (https://gwas.mrcieu.ac.uk/). Based on a previously published article [24], this study selected the aspirin dataset ukb-b-7137 from the European populations, which included 64,534 cases and 393,013 controls. The fracture dataset ebi-a-GCST006980 covered 13,910,100 Europeans (cases = 53,184, controls = 373,611). Five different body site BMD datasets were included, including the femoral neck, lumbar spine, forearm, heel, and ultra distal forearm (Supplementary Table S1).

### Selection of IVs

First, genetic variants significantly associated with the exposure were selected as SNPs, with a significance threshold set at *P* < 5*10^^−8^. Second, strongly linked variants were excluded by setting the parameter R2 to 0.001 and clumping distance to 10,000 kb to reduce the disequilibrium between SNPs. Third, SNPs with a minor allele frequency of less than 0.01 were excluded. Finally, to avoid weak instrument bias, the *F*-values of all SNPs were calculated and only SNPs with *F* > 10 were included [25].

$$F=\frac{\left(\text{N}-2\right)\times R^2}{1-R^2}$$

$$R^2=\frac{2\times\left(1-\mathrm{EAF}\right)\times\mathrm{EAF}\times Beta^2}{2\times\left(1-\mathrm{EAF}\right)\times\mathrm{EAF}\times Beta^2+2\times\left(1-\mathrm{EAF}\right)\times\mathrm{EAF}\times\mathrm{SE}\left(Beta\right)^2\times\mathrm N}$$

Beta represents the estimated genetic effect, EAF denotes the effect allele frequency, N indicates the sample size, and SE(Beta) denotes the standard error of the genetic effect [26].

## Mendelian randomization analysis

### Statistical analysis

Inverse variance-weighted (IVW) was used as the primary method for MR analysis, and the appropriate model was selected based on Cochran’s Q test. A fixed effects model was used by default, and the random effects model was used when heterogeneity was significant (*P* < 0.05). Given that the high accuracy of the IVW method is based on the assumption of no horizontal pleiotropy, this study also introduced MR-Egger and weighted median (WM) as supplementary methods for MR analysis [27, 28]. When significant horizontal pleiotropy exists among IVs, MR-Egger provides the most accurate results [29].

All data were analyzed using the TwoSampleMR package (version 0.5.40) in R (version 4.3.3).

### Sensitivity analysis

Various sensitivity analyses were performed to ensure the reliability of the results. Cochran’s Q test was used to determine the significance of heterogeneity. Leave-one-out analysis and funnel plots were used as part of the sensitivity analysis to ensure the reliability of the results. The Mendelian randomization pleiotropy residual sum and outlier (MR-PRESSO) test was used to detect and remove significant outliers that could bias the results [30]. MR-Egger regression and MR-PRESSO methods were used to assess the significance of horizontal pleiotropy [30].

## Results

### Results of the selection of IVs

The complete specific information on the IVs for aspirin use, bone mineral density, and fractures included in the MR analysis can be found in Supplementary Data S1.

### Effects of aspirin use on BMD and fracture risk

**Fig. 2:**
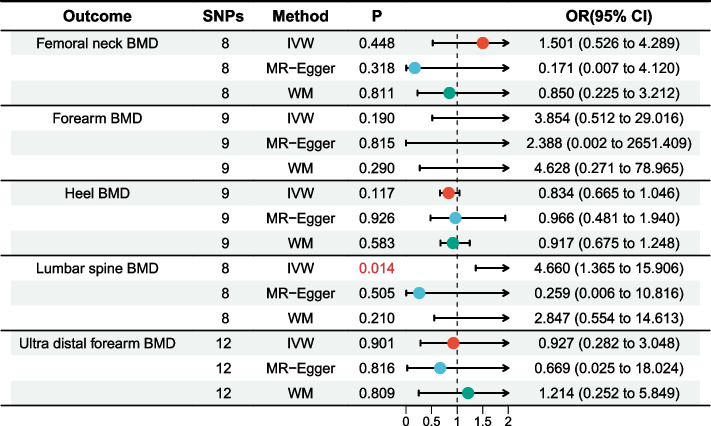
Forest plot of results from Mendelian randomization analysis of aspirin use and bone mineral density

**Fig. 3:**
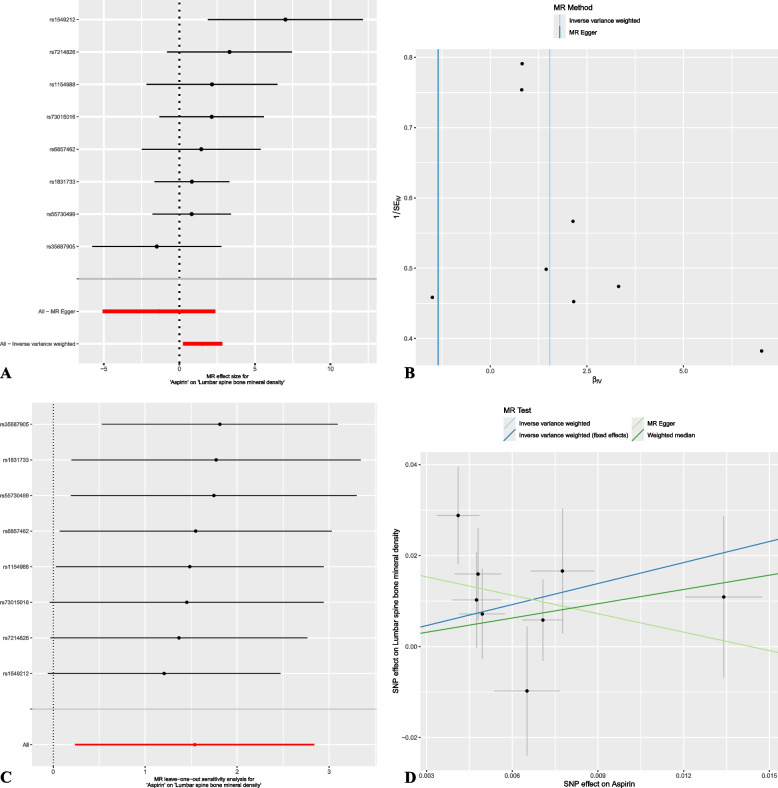
Summary plot of Mendelian randomization analysis between aspirin use and lumbar spine bone mineral density

There was no significant heterogeneity; therefore, a fixed effects model was adopted. MR analysis based on the IVW approach revealed a significant positive correlation between aspirin use and lumbar spine BMD (odds ratio [OR] = 4.660; 95% confidence interval [CI]: 1.365–15.906; *P* = 0.014) (Fig. 2). No significant associations were detected between aspirin use and femoral neck BMD (OR = 1.501; 95% CI: 0.526–4.289; *P* = 0.448), forearm BMD (OR = 3.854; 95% CI: 0.512–29.016; *P* = 0.190), heel BMD (OR = 0.834; 95% CI: 0.665–1.046; *P* = 0.117), or ultra distal forearm BMD (OR = 0.927; 95% CI: 0.282–3.048; *P* = 0.607) (Fig. 2). Similarly, no significant association was found between aspirin use and fracture risk (beta = 59.951; 95% CI: −265.189–385.091; *P* = 0.718). The relationship between aspirin use and lumbar spine BMD was visualized using forest and scatter plots (Fig. 3).

### Effects of BMD and fracture risk on aspirin use

**Fig. 4:**
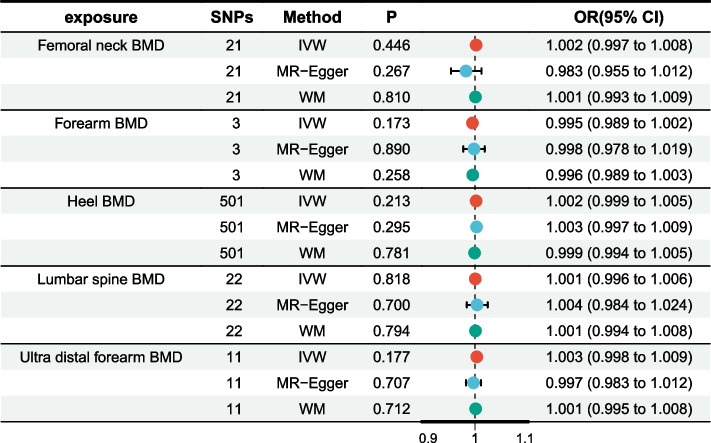
Forest plot of results from reverse Mendelian randomization analysis of aspirin use and bone mineral density

In the reverse MR analysis, no significant associations were detected between the femoral neck, lumbar spine, forearm, heel, or ultra distal forearm BMD and aspirin use (*P* > 0.05) (Fig. 4). Similarly, no significant association was found between fracture risk and aspirin use (*P* = 0.129).

### Results of the sensitivity analysis

For the forward MR analysis of aspirin with respect to BMD and fracture risk, neither the MR Egger nor the MR-PRESSO method detected any significant horizontal pleiotropy (*P* > 0.05), and Cochran’s Q test did not reveal significant heterogeneity (*P* > 0.05) (Supplementary Data S2). Using the IVW method based on a random effects model, a significant positive association between aspirin use and lumbar spine BMD was still observed (OR = 4.660; 95% CI: 1.270–17.104; *P* = 0.020). IVW methods based on fixed effects models and random effects models showed similar results in the remaining MR Analyses (Supplementary Table S2). The pleiotropy and heterogeneity results of MR for fracture risk and BMD with aspirin use are summarized in Supplementary Data S2. Funnel plots and leave-one-out sensitivity analysis results for bidirectional MR analysis of aspirin and lumbar spine BMD are shown in Fig. 3 and Supplementary Fig. 1.

## Discussion

This is the first study to use MR to explore the causal relationship between aspirin use, BMD, and fracture risk from a genetic perspective. This study indicates that aspirin use does not significantly affect fracture risk but has a significant protective effect on lumbar spine bone mineral density.

Animal experiments have shown that aspirin can inhibit osteoclast activation, reduce periosteal reactivity and bone resorption, and promote osteoblast activation and fracture healing [31]. Additionally, aspirin can accelerate the healing of new calli, improve the three-dimensional mechanical structure of trabecular bone, increase bone density, and increase bone biomechanical strength, potentially contributing to the prevention and treatment of osteoporosis [32].

However, no consensus has been reached in previous observational studies on aspirin use and BMD. Attard et al. revealed that individuals taking aspirin for thrombosis prevention had a significantly lower bone mineral density compared to the general population [15]. A cross-sectional survey revealed no association between long-term aspirin use and a decreased BMD [16]. However, more observational studies have concluded that daily aspirin use significantly improves BMD compared with nonaspirin use [10–14], which is consistent with our findings. The conclusions of observational studies on aspirin use and fracture risk are controversial. Vestergaard et al. indicated that aspirin use increases fracture risk [17], whereas multiple observational studies have reported no correlation between aspirin use and fracture risk in the general population [11, 14, 18], which is consistent with our conclusions.

Studies have shown that aspirin may inhibit PGs biosynthesis by acetylating key serine residues in the arachidonic acid binding sites of COX 1 and 2, thereby inhibiting osteoclast activation and promoting osteoblast mineralization involved in bone metabolism regulation [33, 34]. Osteoblasts responsible for bone formation originate from mesenchymal stem cells, whereas osteoclasts responsible for bone resorption originate from hematopoietic stem cells. Pro-inflammatory cytokines such as interleukin-1 and tumor necrosis factor-α (TNF-α) induce COX and nitric oxide synthase to release PGs and NO, contributing to osteoporosis development [35]. Additionally, aspirin inhibits osteoclast formation by reducing receptor activators of nuclear factor kappa-B (NFκB) ligand expression through inhibition of the NFκB pathway and MACK activation in RANKL-induced cells [36, 37]. Furthermore, aspirin promotes the survival and differentiation of osteoblast precursor stem cells and increases osteoprotegerin, thereby inhibiting osteoclast differentiation, and participating in osteoporosis prevention and treatment [36]. Unlike the negative effects observed with high concentrations of aspirin (100 and 150 μg/ml), low-dose aspirin (75 μg/ml) reduces local concentrations of interferon-γ and TNF-α, thereby alleviating inflammatory responses [38]. This anti-inflammatory effect, which inhibits bone erosion and pro-inflammatory mediators, effectively reverses the osteogenic defects induced by pro-inflammatory factors in bone marrow mesenchymal stem cells (BMMSCs), thereby promoting tissue healing [39]. Additionally, the combination of aspirin with osteogenic BFP-1 peptide-modified substrates inhibits the production of pro-inflammatory mediators and promotes osteogenic differentiation of BMMSCs [40]. While low-dose aspirin (< 100 μg/mL), commonly used for daily thrombosis prevention, has been shown to benefit bone mass maintenance and holds potential for OP prevention, high-dose aspirin (150–300 μg/mL) remains ambiguous in its effect on bone metabolism because of its dual action on osteogenesis and bone resorption [41].

The hip and forearm, primarily composed of denser cortical bone, have lower metabolic activity but are crucial regions for reflecting the body’s resistance to fractures. The lumbar spine, predominantly composed of cancellous bone with a higher proportion of trabecular bone and more active bone metabolism, makes lumbar spine BMD a more significant and sensitive indicator of early changes in osteoporosis [42]. This study ultimately identified a positive causal association between aspirin intake and lumbar spine BMD, highlighting the potential protective effect of aspirin against osteoporosis.

This study provides new genetic evidence to address the controversies surrounding observational studies on the association between aspirin intake and osteoporosis. To ensure the reliability of the conclusions, this study strictly adhered to the STROBE-MR guidelines and conducted an MR analysis combined with various sensitivity analyses. BMD data from various skeletal sites were included as extensively as possible to achieve more precise analyses. Some limitations of this study should be acknowledged. Despite previous findings on the differential effects of varying doses of aspirin on bone metabolism, this study was unable to explore the dose–response relationship between aspirin intake, fracture risk, and BMD owing to limitations in dataset information and the MR method. Additionally, this study did not perform stratified analyses based on covariates such as age and sex. Future studies with more robust designs are needed to address these limitations and build on the findings of this study.

## Conclusion

This study indicates that aspirin use does not significantly affect fracture risk but has a significant protective effect on lumbar spine bone mineral density, highlighting the potential benefits of aspirin in osteoporosis prevention and treatment.

## Supplementary Information

Supplementary Material 1: Supplementary Figure S1. Summary plot of reverse Mendelian randomization analysis between aspirin use and lumbar spine bone mineral density.

Supplementary Material 2: Supplementary Table S1. Summary information on the datasets included in the study. Supplementary Table S2. Summary of the results of the MR analysis of the IVW method based on the fixed effects and random effect models.

Supplementary Material 3.

Supplementary Material 4.

## Data Availability

All data utilized in this research were accessible through the IEU OpenGWAS database, which is a publicly accessible platform.

## Abbreviations

BMD: Bone mineral density
MR: Mendelian randomization
COX: Cyclooxygenase
PGs: Prostaglandins
SNPs: Single-nucleotide polymorphisms
IVs: Instrumental variables
IEU: Integrative epidemiology unit
OpenGWAS: Open genome-wide association studies
IVW: Inverse variance-weighted
WM: Weighted median
MR-PRESSO: Mendelian randomization pleiotropy residual sum and outlier
OR: Odds ratio
CI: Confidence interval
TNF-α: Tumor necrosis factor-α
BMMSCs: Bone marrow mesenchymal stem cells
NFκB: Nuclear factor kappa-B
