# Supplementary figures and images for: Application of Mendelian randomization analysis to explore causal associations of aspirin use with bone mineral density and risk of fracture
Source: Hereditas. 2025 Jan 7;162:3. doi: 10.1186/s41065-024-00359-3 (PMC11708298; doi:10.1186/s41065-024-00359-3)

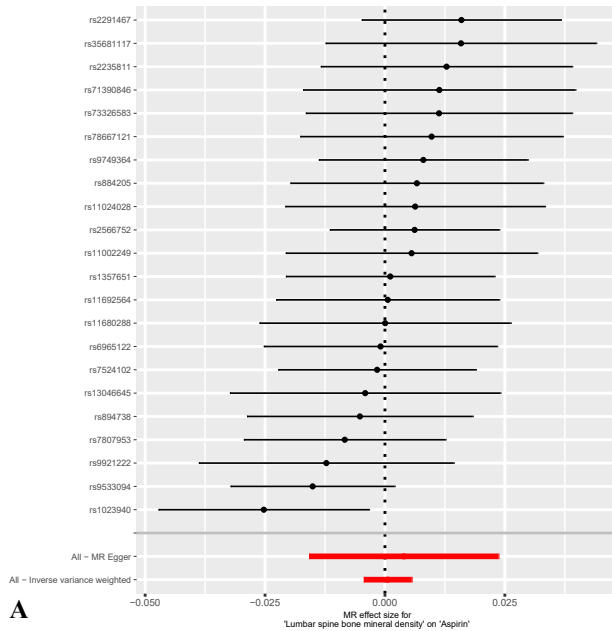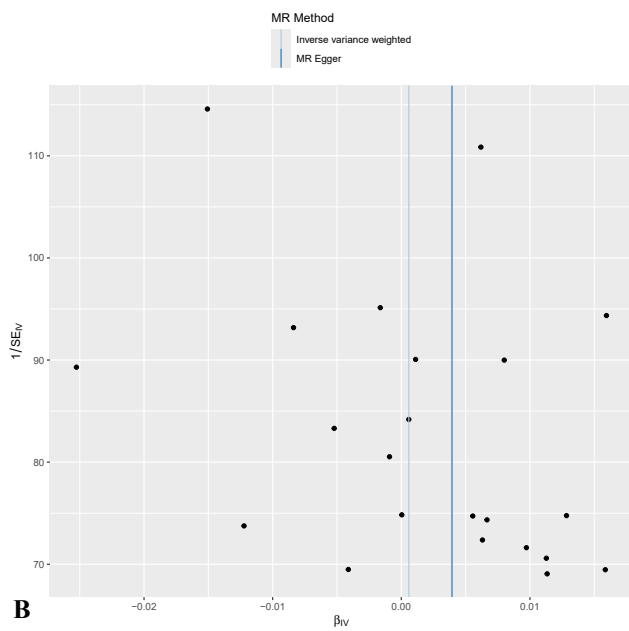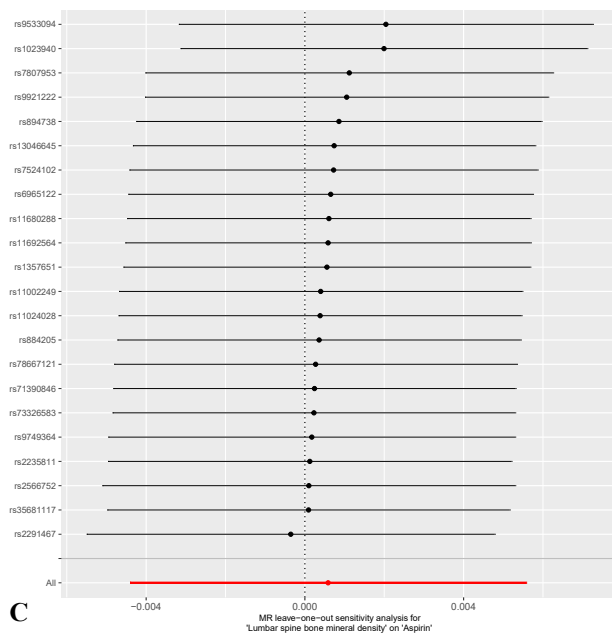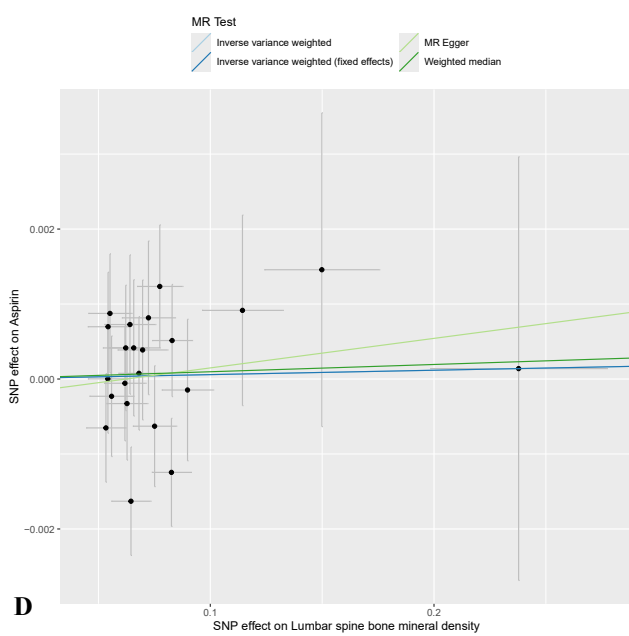

Supplement: Supplementary file 1 — Supplementary Material 1: Supplementary Figure S1. Summary plot of reverse Mendelian randomization analysis between aspirin use and lumbar spine bone mineral density. [file 41065_2024_359_MOESM1_ESM.pdf]
