# Supplementary material for: Application of Mendelian randomization analysis to explore causal associations of aspirin use with bone mineral density and risk of fracture
Source: Hereditas. 2025 Jan 7;162:3. doi: 10.1186/s41065-024-00359-3 (PMC11708298; doi:10.1186/s41065-024-00359-3)
Supplement: Supplementary file 2 — Supplementary Material 2: Supplementary Table S1. Summary information on the datasets included in the study. Supplementary Table S2. Summary of the results of the MR analysis of the IVW method based on the fixed effects and random effect models. [file 41065_2024_359_MOESM2_ESM.docx]

**Application of Mendelian randomization analysis to explore causal associations of aspirin use with bone mineral density and risk of fracture**

Qi-Pei Liu^1*^

1* Guangzhou University of Chinese Medicine, Guangzhou, 510405, China, (e-mail: [lqp67887@163.com](mailto:lqp67887@163.com)).

Supplementary Table S1

Summary information on the datasets included in the study.

| Exposure/Outcome | Consortium | Year | Population | Sample size | Number of SNPs | ID |
| --- | --- | --- | --- | --- | --- | --- |
| Aspirin use | MRC-IEU | 2018 | European | 457,547 | 9,851,867 | ukb-b-7137 |
| Femoral neck bone mineral density | GEFOS | 2015 | Mixed | 32,735 | 10,586,900 | ieu-a-980 |
| Forearm bone mineral density | GEFOS | 2015 | Mixed | 8,143 | 9,955,366 | ieu-a-977 |
| Heel bone mineral density | NA | 2019 | European | 426,824 | 13,705,641 | ebi-a-GCST006979 |
| Lumbar spine bone mineral density | GEFOS | 2015 | Mixed | 28,498 | 10,582,867 | ieu-a-982 |
| Ultra distal forearm bone mineral density | NA | 2020 | European | 21,907 | 11,312,319 | ebi-a-GCST90013422 |
| Fracture | NA | 2019 | European | 426,795 | 13,910,100 | ebi-a-GCST006980 |

Supplementary Table S2

Summary of the results of the MR analysis of the IVW method based on the fixed effects and random effect models.

| Exposure | Outcome | Method | SNPs | OR | OR_95%LCL | OR_95%UCL | P |
| --- | --- | --- | --- | --- | --- | --- | --- |
| Aspirin | Femoral neck BMD | IVW (fixed effects) | 8 | 1.501 | 0.526 | 4.289 | 0.448 |
| Aspirin | Femoral neck BMD | IVW (random effects) | 8 | 1.501 | 0.526 | 4.289 | 0.448 |
| Aspirin | Forearm BMD | IVW (fixed effects) | 9 | 3.854 | 0.512 | 29.016 | 0.190 |
| Aspirin | Forearm BMD | IVW (random effects) | 9 | 3.854 | 0.413 | 35.957 | 0.236 |
| Aspirin | Heel BMD | IVW (fixed effects) | 9 | 0.834 | 0.665 | 1.046 | 0.117 |
| Aspirin | Heel BMD | IVW (random effects) | 9 | 0.834 | 0.640 | 1.087 | 0.180 |
| Aspirin | Lumbar spine BMD | IVW (fixed effects) | 8 | 4.660 | 1.365 | 15.906 | 0.014 |
| Aspirin | Lumbar spine BMD | IVW (random effects) | 8 | 4.660 | 1.270 | 17.104 | 0.020 |
| Aspirin | Ultra distal forearm BMD | IVW (fixed effects) | 12 | 0.927 | 0.282 | 3.048 | 0.901 |
| Aspirin | Ultra distal forearm BMD | IVW (random effects) | 12 | 0.927 | 0.282 | 3.048 | 0.901 |
| Femoral neck BMD | Aspirin | IVW (fixed effects) | 21 | 1.002 | 0.997 | 1.008 | 0.446 |
| Femoral neck BMD | Aspirin | IVW (random effects) | 21 | 1.002 | 0.997 | 1.008 | 0.446 |
| Forearm BMD | Aspirin | IVW (fixed effects) | 3 | 0.995 | 0.989 | 1.002 | 0.173 |
| Forearm BMD | Aspirin | IVW (random effects) | 3 | 0.995 | 0.989 | 1.002 | 0.173 |
| Heel BMD | Aspirin | IVW (fixed effects) | 501 | 1.002 | 0.999 | 1.005 | 0.124 |
| Heel BMD | Aspirin | IVW (random effects) | 501 | 1.002 | 0.999 | 1.005 | 0.213 |
| Lumbar spine BMD | Aspirin | IVW (fixed effects) | 22 | 1.001 | 0.996 | 1.006 | 0.818 |
| Lumbar spine BMD | Aspirin | IVW (random effects) | 22 | 1.001 | 0.996 | 1.006 | 0.818 |
| Ultra distal forearm BMD | Aspirin | IVW (fixed effects) | 11 | 1.003 | 0.998 | 1.009 | 0.177 |
| Ultra distal forearm BMD | Aspirin | IVW (random effects) | 11 | 1.003 | 0.998 | 1.009 | 0.242 |
| Fractures | Aspirin | IVW (fixed effects) | 20 | 1.000 | 1.000 | 1.000 | 0.129 |
| Fractures | Aspirin | IVW (random effects) | 20 | 1.000 | 1.000 | 1.000 | 0.137 |
